# A Vision for 21st Century Agricultural Research

**DOI:** 10.3389/fpls.2012.00157

**Published:** 2012-07-13

**Authors:** Richard A. Jorgensen

**Affiliations:** ^1^Laboratorio Nacional de Genómica para la Biodiversidad, Centro de Investigación y Estudios Avanzados del IPNIrapuato, Guanajuato, México

Let's admit an unfortunate truth: agricultural research in most of the world is based on old models that no longer serve us well in the twenty-first century as arable land diminishes, populations increase, and climate is changing. A dramatically new vision is needed in order to remake agricultural research, perhaps radically, to address the challenges of the world we find ourselves in today. Here is an attempt to begin to develop such a vision.

(1) We must learn to value foundational (basic) and applied research equally. Here are three ways to work toward this goal:
  - Develop the “borderlands” between the Foundational and Applied Research Domains. Recognize and support their unique cultures, while evolving a new culture that bridges the artificial boundary between them.
  - Develop the borderlands between extramural and intramural research within and between government agencies. Value both domains and foster a new culture of cooperation, common mission, and mutual benefits.
  - Develop the borderlands between research and extension. Again, foster a new culture of cooperation, common mission, and mutual benefits.
(2) We need much stronger Foundational Research than currently exists – across the agricultural sciences and in diverse, mission-critical areas that must be defined, prioritized, and supported. Some of these mission-critical areas:
  - Ecosystems, populations, and communities (agricultural and natural).
  - Interorganismal interactions (pathogens, pests, pollinators, mutualisms, etc.).
  - Intraorganismal research: to develop knowledge and understanding leading to *predictive modeling* of growth, development, physiology, and responses to the environment.
  - Environmental and earth sciences, including hydrology, soil science, and remote imaging.
  - Computer and information sciences, mathematical modeling, and cyberinfrastructure in service to, and as a foundation for twenty-first century agricultural sciences: we must learn to *harness the evolving Metaweb* in service to research, extension, and education in order to foster development of a new agriculture based on *intelligent systems*, which are part and parcel of the Metaweb. As described by Nova Spivak (http://www.novaspivack.com/science/new-version-of-my-metaweb-graph-the-future-of-the-net), the Metaweb is continuously evolving from the semantic web and social software, bringing together *knowledge*, and *people* to produce *understanding*.
(3) Applied Science must be reorganized around major concepts, goals, and national and international imperatives, especially:
  - long-term sustainability: production, water, ecosystems, soil quality, farm communities (long-term sustainability may be defined as: “meeting our needs without compromising the ability of future generations to meet their needs”).
  - security: energy security, food security, climate security.
  - health, welfare, and community: nutrition, food quality, robust communities, economic vitality.
(4) To repeat and extend the first point above (because it is so important), success requires that we foster development of *new cultures and new communities that are coincident with needs*, not tradition. The needs for new communities are many and varied. It will be important that new communities must arise out of the borderlands between each of the following pairs of communities that are now largely separate:
  - basic and applied research
  - research and extension
  - agriculture and agroforestry
  - agriculture and ecology
  - agriculture and the food industry
  - the food industry and consumers.

To achieve this vision will require us to let go of habits and preconceived notions in order to work toward *understanding the diverse cultures and communities* that comprise research, extension, and industry and to find new ways of bringing them together productively and creatively in order to create synergies and achieve efficiencies. The specific ways for achieving this vision will be many and varied, and will require the creative efforts of scientists, engineers, funders, industry participants, and policymakers everywhere.

Where should we start? Three principal areas need to be dramatically reorganized: the ways that we fund research, the ways that organize our research institutions, and the ways that we define success and evaluate progress toward the goals above.

Funding priorities need to be dramatically refocused in order to draw researchers out of comfortable isolation into the borderlands where new and challenging interactions between cultures will enable innovative changes, new ways of approaching problems, and disruptive technologies that are needed in order to create a truly sustainable agriculture in sync with sustainable ecosystems and sustainable societies. Probably, the way that funding agencies are organized and funded will need to change in order to make it possible to reorient funding toward new priorities.

Similarly, agricultural research institutions need to be reorganized to bring people together across disciplinary boundaries to populate the borderlands, merge cultures productively, and evolve new cultures and develop new types of institutions. Nothing less than reinventing the nature of agricultural research organizations will suffice, and this reinvention needs to be completed within the next decade if it is to have the needed impact on the necessary timeframe given the increasing demands on agricultural lands, the increasing human population, and changes in climate and health of the ecosystems and the biosphere as a whole.

Finally, of course, none of this can happen unless we also rethink how we evaluate people, programs, and institutions. Expectations and evaluations must be clearly aligned with our new research priorities and the evolving nature of our research organizations. Doing things the way we have always done them is unacceptable if we are to live sustainably for an indefinite time in the only biosphere we have.

